# Case of Quadruplets

**Published:** 1848-07

**Authors:** 


*Case of Quadruplets, by W. W. Welch, M. D.*—We find in the *Illinois and Indiana Medical Journal*, the following extract from a letter by our friend, and quondam pupil, Dr. Welch, addressed to Dr. Brainard, detailing a case of Quadruplets. Dr. W., who has numerous friends in this city, is settled at Inlet, Illinois, and, as we are gratified to learn, is on the high road to professional success, to which his talents and character justly entitle him.—EDITOR BUFFALO MED. JOUR.

Day before yesterday, (20th,) I had just left the house where I had attended a case of childbirth, when I was called in another direction. The woman was but seven months advanced, and my advice was sought rather on the account of extreme anasarca of the legs and thigh, and great œdema of the labia pud., than in expectation of my services being needed obstetrically. She, however, was beginning to have premonitory symptoms of approaching labor, and I disposed myself accordingly; thinking the labia might suffer injury from the passing of the child, I made a number of small punctures with a lancet in each, by which the swelling was reduced, and a large quantity of serum evacuated during the course of labor. The abdomen was enormously enlarged, and the labor was slow, lasting some twenty-four hours. It had been apparently near its termination for several hours, when two fœtuses, in appearance of about six month's growth, were suddenly expelled. One was in a state of incipient putrefaction, and the other barely showed signs of life. Soon after, in searching for the after-birth, my fingers met a very large watery cyst, which as contractions proceeded, was soon ruptured, and an apparently seven months fœtus was delivered by the feet. It was inanimate and there was no pulsation in the cord; but artificial respiration being instituted, it was after a time resuscitated.

Making still another examination after the lapse of a few minutes, the head of a *fourth* child was found to be rapidly advancing, and it was soon in this vale of tears. (I had to laugh, when the woman asked me if I thought that was the last one.) It was more vigorous than the other, and the cord pulsated strongly. The two double placentæ were then extricated; the last adhering slightly, it being necessary to detach it carefully with the fingers.

There was some hemorrage, but it was speedily arrested, and the woman was "comfortable and happy" when I left her yesterday.

The two first born were girls, and the two last boys.

